# Comprehensive chemical profiling of volatile constituents of Angong Niuhuang Pill in vitro and in vivo based on gas chromatography coupled with mass spectrometry

**DOI:** 10.1186/s13020-022-00659-8

**Published:** 2022-09-10

**Authors:** Yue Jiang, Jie Li, Meng Ding, Zi-Fan Guo, Hua Yang, Hui-Jun Li, Wen Gao, Ping Li

**Affiliations:** State Key Laboratory of Natural Medicines, School of Traditional Chinese Pharmacy, China Pharmaceutical University, No. 24 Tongjia Lane, Nanjing, 210009 China

**Keywords:** Angong Niuhuang Pill, Volatile constituents, GC–MS, Pearson correlation coefficient

## Abstract

**Background:**

Angong Niuhuang Pill (ANP), a renowned precious traditional Chinese medicine prescription, is extensively utilized for the clinical treatment of stroke, meningitis and encephalorrhagia in China. As a classic resuscitation-inducing aromatic prescription, ANP has been investigated for its pharmacological effects in recent years, while the volatile composition in ANP still lacks comprehensive elucidation.

**Method:**

To better explore the volatile constituents in ANP, a qualitative analysis method was developed based on gas chromatography coupled with mass spectrometry. Furthermore, a validated quantitative method was established to determine 21 main compounds in 8 batches of commercially available ANP samples by gas chromatography-tandem mass spectrometry. The quantitative data were successively subjected to Pearson correlation coefficient analysis. Additionally, the absorbed volatile constituents in rat plasma after single oral administration of ANP have also been characterized.

**Results:**

A total of 93 volatile constituents including 29 sesquiterpenoids, 28 monoterpenoids, 13 fatty acids and their esters, 7 alkanes, 6 ketones, 3 phenols, 3 aldehydes, 2 benzoate esters, and 2 other types, were preliminarily characterized, which primarily originated from Borneolum, Moschus, Curcumae Radix, and Gardeniae Fructus. d-Borneol, isoborneol and muscone were the top three abundant ingredients (> 600 μg/g) in 8 batches of ANP samples. Subsequently, the average Pearson correlation coefficient of the contents of 21 analytes was 0.993, inferring the high batch-to-batch similarity among 8 batches. After oral administration of ANP, d-borneol, isoborneol, muscone and camphor were the main volatile constituents absorbed in the rat plasma.

**Conclusion:**

This research may be helpful for the comprehensive quality control study of ANP, and provide for guarantee the clinical efficacy of ANP.

**Supplementary Information:**

The online version contains supplementary material available at 10.1186/s13020-022-00659-8.

## Introduction

Angong Niuhuang Pill (ANP), one of the most famous first-aid traditional Chinese medicines (TCMs), contains 11 crude drugs, including Bovis Calculus, Buffalo Horn, Moschus, Margarita, Cinnabaris, Realgar, Coptidis Rhizoma, Scutellariae Radix, Gardeniae Fructus, Curcumae Radix and Borneolum. ANP was first recorded in *Treatise on Differentiation and Treatment of Epidemic Febrile Diseases* in the Qing Dynasty and has a history of over 200 years of application in China. According to the TCM theory and clinical practice, ANP has been proved to be beneficial to the treatment of various central nervous system diseases, such as stroke coma, meningitis and intracerebral hemorrhage [1]. A recent meta-analysis of 18 trials involving 1601 patients reported that adjuvant treatment with ANP could significantly improve the total response rate and reduce the neurologic deficit score in patients with acute cerebral infarction and acute intracerebral hemorrhage [2].

Generally, ANP contains volatile and non-volatile constituents, contributing to an integral part of the overall efficacy of ANP. The volatile substances of aromatic drugs are mainly deemed to process the important properties of resuscitation and awakening in traditional Chinese medicines. Since ANP is a typical resuscitation-inducing aromatic prescription, its therapeutic effect on cerebrovascular diseases may be closely related to the volatile components. For instance, muscone, d-borneol, and isoborneol, the representative components from Moschus, Borneolum, respectively, showed various biological activities in vitro and in vivo. Muscone might treat myocardial infarction and protect cardiovascular and cerebrovascular system [3, 4], regulate neuroprotective system [5] and improve osteoarticular injuries [6]. Isoborneol and d-borneol had the characteristics of anti-inflammatory [7], anti-atherosclerosis [8] and promoting penetration [9], and played significant roles in the treatment of cerebrovascular diseases [12]. However, there are few researches on systematical characterization the volatile chemical composition in ANP.

In this study, the volatile constituents in ANP were comprehensively characterized based on the sensitive gas chromatography coupled with mass spectrometry (GC–MS) in vitro and in vivo. The GC–MS method was complemented for the volatile compounds identification based on database matching, retention indices and standard references confirmation. Then, 21 compounds were quantified in 8 batches of ANP by gas chromatography-tandem mass spectrometry (GC–MS/MS), and the batch-to-batch similarity was evaluated by Pearson correlation coefficient (PCC) analysis. Furthermore, the volatile constituent of ANP in vivo were characterized in the plasma of rats which was single oral administrated. To our knowledge, the determination of main volatile compounds in ANP was reported for the first time. It is hoped that the results can provide a valuable reference for quality control and clinical efficacy research of ANP.

## Methods

### Reagents and materials

Eight batches of ANP (No. S1–S8) were purchased from Beijing TongRenTang Technologies Co., Ltd and their information was listed in Additional file 1: Table S1. All the ANP samples used artificial Moschus and in vitro cultured Bovis Calculus in their prescription. The reference standards of muscone, α-pinene, limonene, eucalyptol, isoborneol, and d-borneol were obtained from National Institutes for Food and Drug Control (Beijing, China). Acetophenone, fenchol and β-caryophyllene were bought from Shanghai Yuanye Bio-Technology Co., Ltd (Shanghai, China). Camphene, benzaldehyde, α-terpinene, benzeneacetaldehyde, terpinolene, camphor, tridecane, 4-methyl-4-phenyl-2-pentanone, and humulene were acquired from Chengdu Push Bio-Technology Co., Ltd (Chengdu, China). ar-Tumerone was purchased from BioBioPha Co., Ltd. (Kunming, China). The standard mix of n-alkanes (C7–C40) was obtained from Sigma-Aldrich (St. Louis, MO, USA). (+)-3-Carene was bought from Toronto Research Chemicals (Toronto, ON, Canada). β-Pinene was acquired from Dr. Ehrensdorfer GmbH (Augsburg, Germany). α-Curcumene was purchased from Extrasynthese (Lyon Nord, France). The purities of all used reference standards were higher than 95%.

Anhydrous ethanol and ethyl acetate (HPLC grade) were purchased from Yonghua Chemical Technology Co., Ltd (Suzhou, China). Distilled water was prepared using a Milli-Q Integral water purification system (Millipore, Bedford, MA, USA). Other reagents were analytical grade.

### Preparation of standard and sample solutions

The 21 reference standards were dissolved with anhydrous ethanol respectively to prepare the corresponding stock solutions at the concentration of 1 mg/mL. An appropriate amount of individual standard stock solutions was mixed into a 10 mL volumetric flask to prepare a mixed standard stock solution, which was diluted with anhydrous ethanol to obtain a series of working solutions at proper concentrations for calibration curves. All working solutions were stored at − 20 °C until analysis.

After ground into fine powder, 1.5 g ANP was accurately weighed and extracted using steam distillation for 4 h with 100 mL distilled water. The volatile oils were collected and residual water was removed with anhydrous sodium sulfate, finally were dissolved in 2 mL ethyl acetate. The solution was directly analyzed after adding n-tridecane as an internal standard (IS). Since some compounds of high contents such as isoborneol, d-borneol, β-caryophyllene, ar-turmerone and muscone were overloaded, the solution was also analyzed after diluted by 40 times with ethyl acetate and added with IS. The n-tridecane in all injection samples was at the final concentration of 14.20 μg/mL.

### Collection and precipitation procedure of plasma samples

Male Wistar rats (Animal certificate number: SCXK [HU]-2018-0016) were purchased from Shanghai Lab. Animal Research Center (Shanghai, China). All rats were housed at 24 ± 2 °C on a 12 h light/dark cycle, and fed a standard diet and water for 1 week before the experiment. Then all rats (200 ± 20 g) were randomly divided into two groups: ANP group and control group. The rats were fasted for 12 h prior to experiments but water was provided ad libitum. ANP suspension dissolved in physiological saline was orally administrated to nine rats (ANP group) at a dosage of 8.1 g/kg, and the rest two rats (control group) were orally administered with the same dose of saline respectively. All procedures were carried out in accordance with Guide for the Care and Use of Laboratory Animals (National Institutes of Health).

The blood samples were collected in heparinized 1.5-mL polythene tubes at 0, 0.25, 0.5, 0.75, 1, 1.5, 2 and 4 h after oral administration. At each time point, the blood samples of 3 rats (ANP group) or 2 rats (control group) were mixed into one sample, and were centrifuged immediately at 4500 rpm for 10 min at 4 °C to collect the plasma, which were stored at − 80 °C before analysis. For GC–MS analysis, a 100 μL aliquot of plasma sample was added with 100 μL of ethyl acetate, followed by vortex-mixing for 1 min and centrifugation for 10 min at 13,000 rpm. An aliquot of supernatant was transferred into an injection vial for analysis.

### Chromatographic and mass spectrometric conditions

The qualitative analysis was performed on an Agilent 7890B gas chromatography system coupled to an Agilent 5977A quadrupole mass spectrometer (GC–MS, Agilent Technologies, USA). The GC separation was achieved on an Agilent DB-5 MS capillary column (60 m × 0.25 mm i.d.) coated with a 0.25 μm film of 5% phenyl polymethyl siloxane. High-purity helium gas was used as carrier gas, with a flow rate of 1.0 mL/min. The injection and interface temperatures were set to 280 °C and 250 °C, respectively. The column temperature was programmed as follows: the oven was initially maintained at 60 °C for 3 min, sequentially increased to 100 °C at 10 °C/min, increased to 135 °C at 2 °C/min, then increased to 165 °C at 5 °C/min, increased to 168 °C at 1 °C/min, increased to 200 °C at 5 °C/min, held for 2 min, and then increased to 295 °C at 10 °C/min, held constant for 5 min. The split ratio was set to 10:1. The injection volume was 1 μL. The electron energy was set to 70 eV. The source temperature was 230 °C and the quadrupole temperature was 150 °C. The mass data was acquired using full scan mode with a mass range of *m/z* 50–600 after a solvent delay of 9.1 min. Data acquisition was obtained by Agilent MassHunter GC–MS Acquisition Software Version B.07.03.2129.

For quantitative analysis, the Agilent 7890B GC system equipped with an Agilent 7000D triple quadrupole mass spectrometer (GC–MS/MS, Agilent Technologies, USA) was performed. The Agilent DB-5 MS UI capillary column (30 m × 0.25 mm i.d., 0.25 μm) were used for separation, the initial oven temperature was 60 °C, and raised to 76 °C with 8 °C/min, then increased to 82 °C at the rate of 2 °C/min, and ramped with 15 °C/min to 130 °C, further rose at 20 °C/min to 230 °C and held for 2 min. The injection and interface temperatures were both kept at 250 °C. The carrier gas (helium, > 99.999%) flow rate was set at 1.0 mL/min. The electron energy and source temperature were set to 70 eV and 230 °C, respectively. The flow rate of quenching gas (helium, > 99.999%) and collision gas (nitrogen, > 99.999%) was 2.25 mL/min and 1.5 mL/min, separately. Data acquisition was achieved on Agilent MassHunter Workstation GC/MS Data Acquisition Software Version 10.0.368.

### Validation of quantitative method

#### Calibration curves, LODs and LOQs

After added with an equal amount of internal standard (IS) *n*-tridecane (final concentration: 14.20 μg/mL), a series of working solutions at multiple concentrations were analyzed. The calibration curves were constructed by plotting the relationships between peak area and concentration of the analytes and IS.

The mixed standard stock solution was further diluted with anhydrous ethanol and analyzed by GC–MS/MS. The concentration of the analyte with signal-to-noise ratio (*S/N*) of 10 was defined as the limit of quantification (LOQ), and the concentration of the analyte with *S/N* of 3 was assigned as the limit of detection (LOD).

#### Precision, repeatability, stability and recovery

The precision was evaluated by the determination of intra- and inter-day variances. The mixed standard solutions at three different concentration levels (low, medium, high) were analyzed for consecutive 3 days and continuous six times per day, and the peak area ratio of each compound and IS was recorded to calculate the relative standard deviation (RSD) value, respectively. Six parallel ANP samples from the same batch were prepared and analyzed for the repeatability test. To confirm the stability, a single sample solution was stored in sample chamber and analyzed at 0, 2, 4, 8, 12 and 24 h, respectively. Recovery test was used to verify the accuracy of the established method. A known amount of mixed reference solution at middle concentration was added in the same sample for six parallel extraction and analysis.

### Data analysis

The qualitative analysis was realized on Agilent MassHunter Workstation Qualitative Analysis Software 10.0. The retention indices of all chromatographic peaks were calculated based on the data of C7–C40 *n*-alkanes acquired with the same GC–MS method. The acquired components were tentatively identified by comparison with mass spectra and retention indices in the National Institute of Standards and Technology (NIST) 2017 library, and some were unambiguously determined by direct comparison with reference standards. Agilent MassHunter Workstation Quantitative Analysis Software Version 10.0 was used for quantitative analysis. Related graphical analysis was conducted on Graphpad Prism 8.02 (San Diego, USA).

The pairwise PCC between batches was based on the vectors of concentrations from each batch as Formula (1).

1 $$PCC_{1,2} = \frac{{\sum\nolimits_{i = 1}^{n} {\left( {B1_{i} - \overline{B}1} \right)\left( {B2_{i} - \overline{B}2} \right)} }}{{\sqrt {\sum\nolimits_{i = 1}^{n} {\left( {B1_{i} - \overline{B}1} \right)^{2} } } \sqrt {\sum\nolimits_{i = 1}^{n} {\left( {B2_{i} - \overline{B}2} \right)^{2} } } }}.$$

Here, B1 and B2 represented the vectors of ingredients’ concentration of the first and second batches respectively. Finally, the PCCs were displayed through the correlation heat map, the batch-to-batch similarity was evaluated according to the average value of PCCs among all pairs from batches.

## Results and discussion

### Characterization of volatile constituents in ANP

**Fig. 1:**
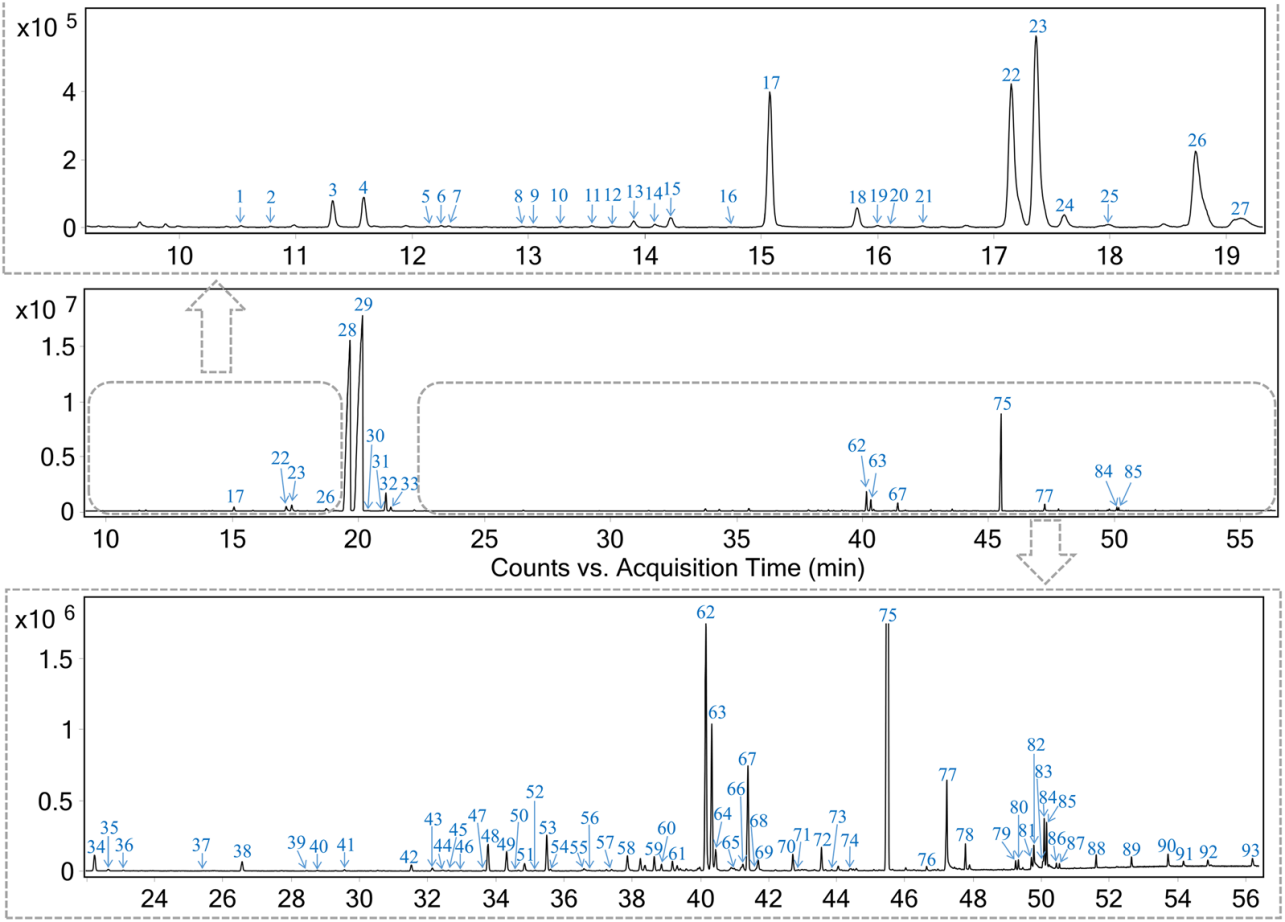
The total ion chromatogram of the essential oil from Angong Niuhuang Pill based on GC–MS

For the qualitative analysis, the temperature-programmed conditions of GC–MS were optimized in terms of resolution, symmetricity and capacity of chromatographic peaks. Then, the mixed reference standard solution and ANP sample solution were analyzed by the optimized GC–MS method, and the total ion chromatogram of the essential oil from ANP is shown in Fig. 1. The volatile constituents of ANP were identified by comparing their mass spectra and retention indices with those of NIST library, and accurately identified by matching with authentic standards. As illustrated in Fig. 1 and Table 1, a total of 93 components, including 29 sesquiterpenoids, 28 monoterpeneoids, 13 fatty acids and their esters, 7 alkanes, 6 ketones, 3 phenols, 3 aldehydes, 2 benzoate esters and 2 other type components, were preliminarily characterized. Among them, 21 compounds were determined by comparison with reference standards. The essential oil was dominated by d-borneol, isoborneol and muscone, making up 90.69% of the identified oil composition.

**Table 1:** Identification of the chemical constituents in the essential oil of ANP by GC–MS

No.	t_R_ (min)	Compound	Formula	CAS	RI (measured)	RI (NIST)	Match	Structure type	Source
1	10.53	Tricyclene	C_10_H_16_	508-32-7	930.02	925	853	Monoterpeneoids	B
2	10.79	α-Pinene^a^	C_10_H_16_	80-56-8	939.35	937	872	Monoterpeneoids	GF, CR
3	11.32	Camphene^a^	C_10_H_16_	79-92-5	958.17	952	954	Monoterpeneoids	B, CR
4	11.59	Benzaldehyde^a^	C_7_H_6_O	100-52-7	967.74	962	951	Aldehydes	GF
5	12.13	β-Pinene^a^	C_10_H_16_	127-91-3	987.29	979	847	Monoterpeneoids	CR
6	12.25	2-Pentylfuran	C_9_H_14_O	3777-69-3	991.45	993	839	Furans	GF
7	12.32	2,2,4,6,6-Pentamethylheptane	C_12_H_26_	13475-82-6	993.74	991	897	Alkanes	B
8	12.95	α-Phellandrene	C_10_H_16_	99-83-2	1012.46	1005	906	Monoterpeneoids	CR
9	13.05	(+)-3-Carene^a^	C_10_H_16_	498-15-7	1015.16	1010	740	Monoterpeneoids	CR
10	13.29	α-Terpinene^a^	C_10_H_16_	99-86-5	1021.92	1017	825	Monoterpeneoids	CR
11	13.54	*p*-Cymene	C_10_H_14_	99-87-6	1028.7	1025	900	Monoterpeneoids	GF, CR
12	13.72	Limonene^a^	C_10_H_16_	138-86-3	1033.6	1030	836	Monoterpeneoids	GF, CR
13	13.9	Eucalyptol^a^	C_10_H_18_O	470-82-6	1038.53	1032	934	Monoterpeneoids	GF, CR
14	14.09	β-Isophorone	C_9_H_14_O	471-01-2	1043.5	1044	843	Ketones	GF
15	14.22	Benzeneacetaldehyde^a^	C_8_H_8_O	122-78-1	1047.2	1045	935	Aldehydes	GF
16	14.73	r-Terpinene	C_10_H_16_	99-85-4	1061.04	1060	733	Monoterpeneoids	CR
17	15.07	Acetophenone^a^	C_8_H_8_O	98-86-2	1070.44	1065	965	Ketones	GF
18	15.83	Terpinolene^a^	C_10_H_16_	586-62-9	1090.9	1088	935	Monoterpeneoids	CR
19	16.01	*p*-Cymenene	C_10_H_12_	1195-32-0	1095.82	1090	843	Monoterpeneoids	CR
20	16.1	Fenchone	C_10_H_16_O	1195-79-5	1098.36	1096	810	Monoterpeneoids	B
21	16.39	Nonanal	C_9_H_18_O	124-19-6	1105.11	1104	872	Aldehydes	GF
22	17.15	Isofenchol	C_10_H_18_O	6168-62-3	1121.75	–	918	Monoterpeneoids	B
23	17.37	Fenchol^a^	C_10_H_18_O	1632-73-1	1126.45	1113	904	Monoterpeneoids	B
24	17.61	β-Fenchol	C_10_H_18_O	22627-95-8	1131.74	1116	848	Monoterpeneoids	B
25	17.98	α-Campholenal	C_10_H_16_O	4501-58-0	1140.03	1125	828	Monoterpeneoids	B
26	18.74	Camphor^a^	C_10_H_16_O	76-22-2	1156.5	1145	930	Monoterpeneoids	B
27	19.12	Camphene hydrate	C_10_H_18_O	465-31-6	1164.88	1148	925	Monoterpeneoids	B
28	19.67	Isoborneol^a^	C_10_H_18_O	124-76-5	1177.11	1157	976	Monoterpeneoids	B
29	20.17	d-Borneol^a^	C_10_H_18_O	464-43-7	1188.07	1167	963	Monoterpeneoids	B
30	20.39	α,α,3-Trimethylbenzenemethanol	C_10_H_14_O	5208-37-7	1192.7	1180	845	Alcohols	CR
31	20.83	l-α-Terpineol	C_10_H_18_O	10482-56-1	1202.58	1190	819	Monoterpeneoids	GF, CR
32	21.11	2-Bornanol	C_10_H_18_O	10,385-78-1	1207.45	–	811	Monoterpeneoids	B
33	21.3	Neoisopulegol	C_10_H_18_O	29141-10-4	1211.19	–	813	Monoterpeneoids	B
34	22.22	4-Methyleneisophorone	C_10_H_14_O	20548-00-9	1229.01	1242	952	Ketones	GF
35	22.64	Bornyl formate	C_11_H_18_O_2_	7492-41-3	1236.94	1226	908	Monoterpeneoids	B
36	23.08	Isobornyl formate	C_11_H_18_O_2_	1200-67-5	1245.47	1232	818	Monoterpeneoids	B
37	25.41	l-Bornyl acetate	C_12_H_20_O_2_	5655-61-8	1290.31	1284	640	Monoterpeneoids	B
38	26.55	4-Vinylguaiacol	C_9_H_10_O_2_	7786-61-0	1314.47	1317	932	Phenols	CR
39	28.45	Eugenol	C_10_H_12_O_2_	97-53-0	1357.5	1357	819	Phenols	GF
40	28.76	Benzalacetone	C_10_H_10_O	122-57-6	1364.59	–	865	Ketones	GF
41	29.56	4-Methyl-4-phenyl-2-pentanone^a^	C_12_H_16_O	7403-42-1	1382.7	–	769	Ketones	CR
42	31.52	β-Caryophyllene^a^	C_15_H_24_	87-44-5	1429.53	1419	932	Sesquiterpenoids	CR
43	32.13	Paeonol	C_9_H_10_O_3_	552-41-0	1444.74	1438	914	Phenols	GF
44	32.44	*trans*-β-Farnesene	C_15_H_24_	18794-84-8	1452.39	1457	796	Sesquiterpenoids	CR
45	32.61	Sesquisabinene	C_15_H_24_	58319-04-3	1456.51	1464	811	Sesquiterpenoids	CR
46	32.98	Humulene^a^	C_15_H_24_	6753-98-6	1465.58	1454	804	Sesquiterpenoids	CR
47	33.65	1,3-Cyclohexadiene, 1-(1,5-dimethyl-4-hexenyl)-4-methyl-	C_15_H_24_	451-55-8	1482.09	1480	876	Sesquiterpenoids	CR
48	33.77	α-Curcumene^a^	C_15_H_22_	644-30-4	1485.18	1483	957	Sesquiterpenoids	CR
49	34.32	α-Zingiberene	C_15_H_24_	495-60-3	1498.67	1495	891	Sesquiterpenoids	CR
50	34.57	α-Farnesene	C_15_H_24_	502-61-4	1505.36	1508	672	Sesquiterpenoids	CR
51	34.84	β-Bisabolene	C_15_H_24_	495-61-4	1513.02	1509	919	Sesquiterpenoids	CR
52	35.15	Ethylparaben	C_9_H_10_O_3_	120–47-8	1521.51	–	910	Benzoate esters	M
53	35.49	β-Sesquiphellandrene	C_15_H_24_	20307-83-9	1530.9	1524	929	Sesquiterpenoids	CR
54	35.61	*trans*-γ-Bisabolene	C_15_H_24_	53585-13-0	1534.09	1533	833	Sesquiterpenoids	CR
55	36.59	*cis*-Sesquisabinene hydrate	C_15_H_26_O	58319-05-4	1561.06	1543	857	Sesquiterpenoids	CR
56	36.72	*trans*-Nerolidol	C_15_H_26_O	40716-66-3	1564.79	1564	750	Sesquiterpenoids	CR
57	37.39	ar-Tumerol	C_15_H_22_O	38142-57-3	1583.26	1583	792	Sesquiterpenoids	CR
58	37.86	*trans*-Sesquisabinene hydrate	C_15_H_26_O	145,512–84-1	1596.08	1581	900	Sesquiterpenoids	CR
59	38.64	Zingiberenol	C_15_H_26_O	58334-55-7	1620.64	1616	923	Sesquiterpenoids	CR
60	38.85	*trans*-Nuciferol	C_15_H_22_O	39599-18-3	1627.39	–	712	Sesquiterpenoids	CR
61	39.18	β-Acorenol	C_15_H_26_O	28400-11-5	1637.839241	1649	808	Sesquiterpenoids	CR
62	40.16	ar-Tumerone^a^	C_15_H_20_O	532-65-0	1669.3	1664	949	Sesquiterpenoids	CR
63	40.33	Tumerone	C_15_H_22_O	180315-67-7	1674.84	1632	935	Sesquiterpenoids	CR
64	40.44	(*Z*)-γ-Atlantone	C_15_H_22_O	108549-48-0	1678.45	1699	836	Sesquiterpenoids	CR
65	41	4-(1,5-Dimethylhex-4-enyl)cyclohex-2-enone	C_14_H_22_O	1723-80-4	1696.24	1698	848	Sesquiterpenoids	CR
66	41.24	Bisacurol	C_15_H_24_O	120681-80-3	1704.32	–	910	Sesquiterpenoids	CR
67	41.4	Curlone	C_15_H_22_O	82508-14-3	1709.55	–	948	Sesquiterpenoids	CR
68	41.61	Curcuphenol	C_15_H_22_O	69301-27-5	1716.86	1717	814	Sesquiterpenoids	CR
69	41.7	(*Z*)-α-Atlantone	C_15_H_22_O	56192-70-2	1719.91	1717	915	Sesquiterpenoids	CR
70	42.71	(6*R*,7*R*)-Bisabolone	C_15_H_24_O	72441-71-5	1754.45	1747	936	Sesquiterpenoids	CR
71	42.82	Tetradecanoic acid	C_14_H_28_O_2_	544-63-8	1758.18	1768	780	Fatty acids and their esters	GF
72	43.55	(−)-(*E*)-α-Atlantone	C_15_H_22_O	108645-54-1	1783.13	1773	927	Sesquiterpenoids	CR
73	43.85	Ethyl myristate	C_16_H_32_O_2_	124-06-1	1793.15	1794	665	Fatty acids and their esters	GF
74	44.39	Turmeronol B	C_15_H_20_O_2_	131651-38-2	1815.66	–	763	Sesquiterpenoids	CR
75	45.5	Muscone^a^	C_16_H_30_O	541-91-3	1867.53	–	929	Ketones	M
76	46.64	Methyl palmitate	C_17_H_34_O_2_	112-39-0	1925.78	1926	868	Fatty acids and their esters	M, GF
77	47.23	Palmitic acid	C_16_H_32_O_2_	10/3/1957	1960.36	1968	957	Fatty acids and their esters	M, GF
78	47.78	Ethyl palmitate	C_18_H_36_O_2_	628-97-7	1992.48	1993	915	Fatty acids and their esters	B, M, GF, CR
79	49.25	Methyl linoleate	C_19_H_34_O_2_	112-63-0	2094.97	2092	904	Fatty acids and their esters	GF
80	49.33	Methyl oleate	C_19_H_36_O_2_	112-62-9	2100.76	2091	846	Fatty acids and their esters	M, GF
81	49.72	Linoleic acid	C_18_H_32_O_2_	60-33-3	2132.12	2133	900	Fatty acids and their esters	M, GF
82	49.79	Oleic acid	C_18_H_34_O_2_	112–80-1	2138.03	2141	887	Fatty acids and their esters	M, GF
83	50.05	Stearic acid	C_18_H_36_O_2_	11/4/1957	2159.94	2172	842	Fatty acids and their esters	M, GF
84	50.09	Ethyl linoleate	C_20_H_36_O_2_	544-35-4	2163.23	2162	937	Fatty acids and their esters	M
85	50.17	Ethyl oleate	C_20_H_38_O_2_	111-62-6	2169.06	2173	905	Fatty acids and their esters	M
86	50.45	Ethyl stearate	C_20_H_40_O_2_	111-61-5	2192.34	2195	811	Fatty acids and their esters	B
87	50.54	Docosane	C_22_H_46_	629-97-0	2199.89	2200	855	Alkanes	GF
88	51.62	Tricosane	C_23_H_48_	638-67-5	2299.86	2300	880	Alkanes	GF
89	52.65	Tetracosane	C_24_H_50_	646-31-1	2399.86	2400	886	Alkanes	GF
90	53.72	Pentacosane	C_25_H_52_	629-99-2	2499.78	2500	878	Alkanes	GF
91	54.17	Di(2-propylpentyl) phthalate	C_24_H_38_O_4_	70910-37-1	2538.21	2527	911	Benzoate esters	GF
92	54.89	Hexacocane	C_26_H_54_	630-01-3	2599.7	2600	873	Alkanes	GF
93	56.2	Heptacosane	C_27_H_56_	593-49-7	2699.46	2700	851	Alkanes	GF

The batch of ANP used for qualification was S7

Sources: *B* Borneolum, *M* Moschus, *CR* Curcumae Radix, *GF* Gardeniae Fructus

–: The RI was not found in NIST library

^a^The identification was confirmed with reference standards

Among the 93 preliminarily identified volatile compounds, 11 components might be derived from Moschus, including 1 ketone, 1 benzoate ester and 9 fatty acids and their esters. The most abundant composition in Moschus was muscone (Peak **75**) [13], which was also one of the principal constituents in ANP. Furthermore, 19 compounds comprising 16 monoterpeneoids, 2 fatty acid esters and 1 alkane, were tentatively assigned to Borneolum, of which the highest were d-borneol (Peak **29**) and isoborneol (Peak **28**) [7]. Additionally, 46 components were originated from Curcumae Radix, consisting of 29 sesquiterpenoids, 13 monoterpeneoids, 1 phenol, 1 ketone, 1 fatty acid ester, and 1 alcohol. Chinese Pharmacopoeia recorded its four botanical origins, including *Curcuma wenyujin* Y. H. Chen et C. Ling, *Curcuma longa* L., *Curcuma phaeocaulis* Val. and *Curcuma kwangsiensis* S. G. Lee et C. F. Liang. Sesquiterpenoid was one of the most abundant types in Curcumae Radix. Baesd on the characteristic constituents including ar-Tumerone (Peak **62**), β-caryophyllene (Peak **42**) and α-curcumene (Peak **48**), the Curcumae Radix in those ANP could be traced to *Curcuma longa* L. [14–16]. Furthermore, 32 volatile components in ANP could be sourced from Gardeniae Fructus, containing 10 fatty acids and their esters, 6 alkanes, 5 monoterpeneoids, 4 ketones, 3 aldehydes, 2 phenols, and 1 furan and 1 benzoate ester. The constituents containing “-cyclohexane-trimethyl-” group represented by 4-methyleneisophorone (Peak **34**) and β-Isophorone (Peak **14**) might be the characteristic aromatic compounds in Gardeniae Fructus [17], which were detected in ANP. The detailed sources of volatile constituents in ANP are shown in Table 1 and Additional file 2: Fig. S1.

### Quantitative analysis of representative volatile constituents in ANP

**Fig. 2:**
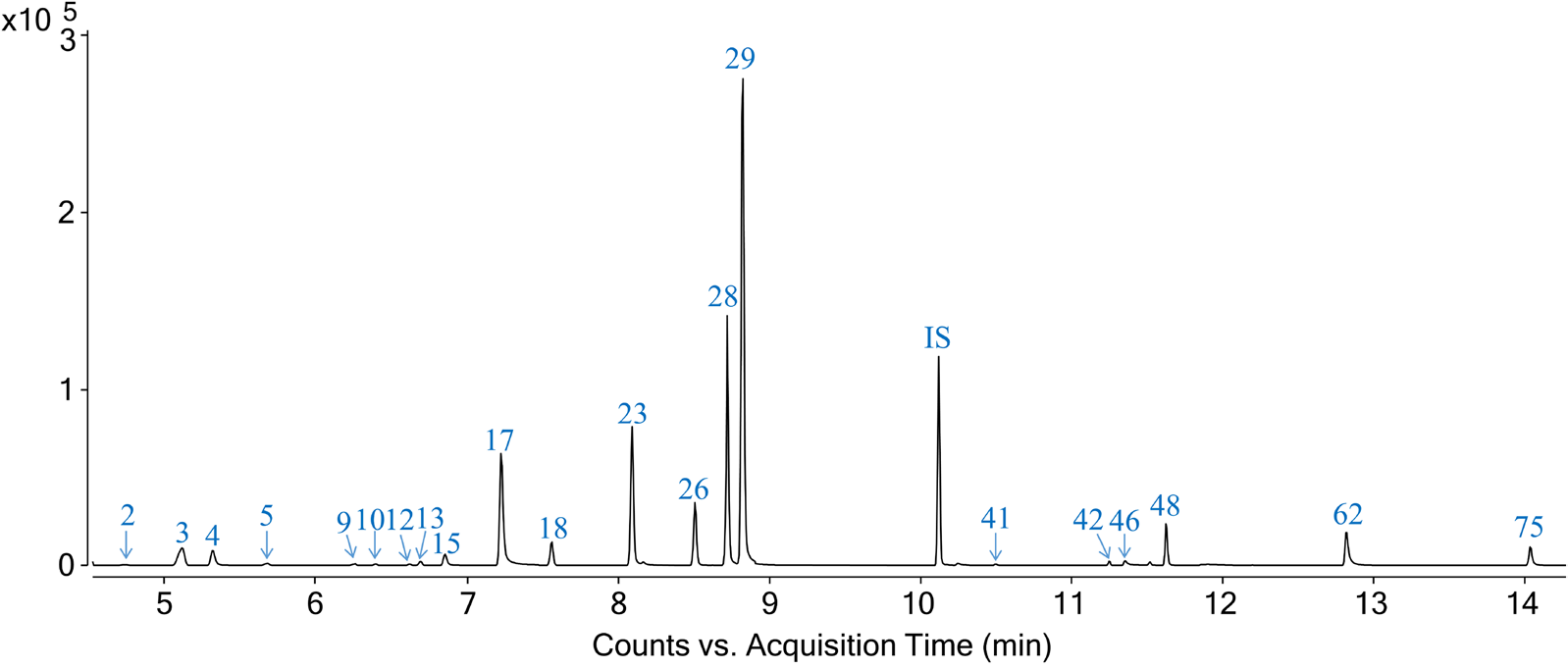
The segmented dMRM chromatogram of 21 target compounds and internal standard based on GC–MS/MS

For the quantitative analysis, a temperature-programmed process of GC–MS/MS for 21 volatile constituents was developed within 15 min. A dynamic multiple reaction monitoring (dMRM) pattern was selected for quantification. As an alternative for multiple reaction monitoring, dMRM can monitor analytes automatically around the expected retention time, without defining a specific time period for the selected transitions, thereby decreasing concurrent transitions and improving the sensitivity [18, 19]. The segmented dMRM chromatogram of 21 target compounds is shown in Fig. 2. The dMRM parameters for all analytes and IS are presented in Additional file 1: Table S2.

#### Method validation

The results of methodological study are shown in Additional file 1: Tables S3, S4. For the calibration curve established, all analytes showed good linearity within their linear ranges and their correlation coefficient R^2^ values varied from 0.9918 to 0.9997. The LODs and LOQs ranged from 0.60 to 150.40 ng/mL and from 6.00 to 451.20 ng/mL, respectively, indicating this method was sensitive enough for the quantitative analysis of the major volatile constituents of ANP. The RSD values of the intra- and inter-day were in the ranges of 0.04–5.84% and 0.48–6.83%, respectively. The RSD values for repeatability of all compounds ranged from 1.13 to 9.00%, which showed that the repeatability of tested samples was good. The RSD values for stability of all analytes were with the range of 1.39–6.70%, indicating that the samples in sample chamber were relatively stable within 24 h. The overall recoveries of 21 compounds were in a range of 90.80–109.83% with RSD values less than 8.68%, which indicated the verified method was accurate for quantification. The results demonstrated that the developed quantitative method was sensitive, rapid, accurate and reproducible for determination of representative volatile constituents in ANP.

#### Contents of representative volatile constituents in ANP

**Fig. 3:**
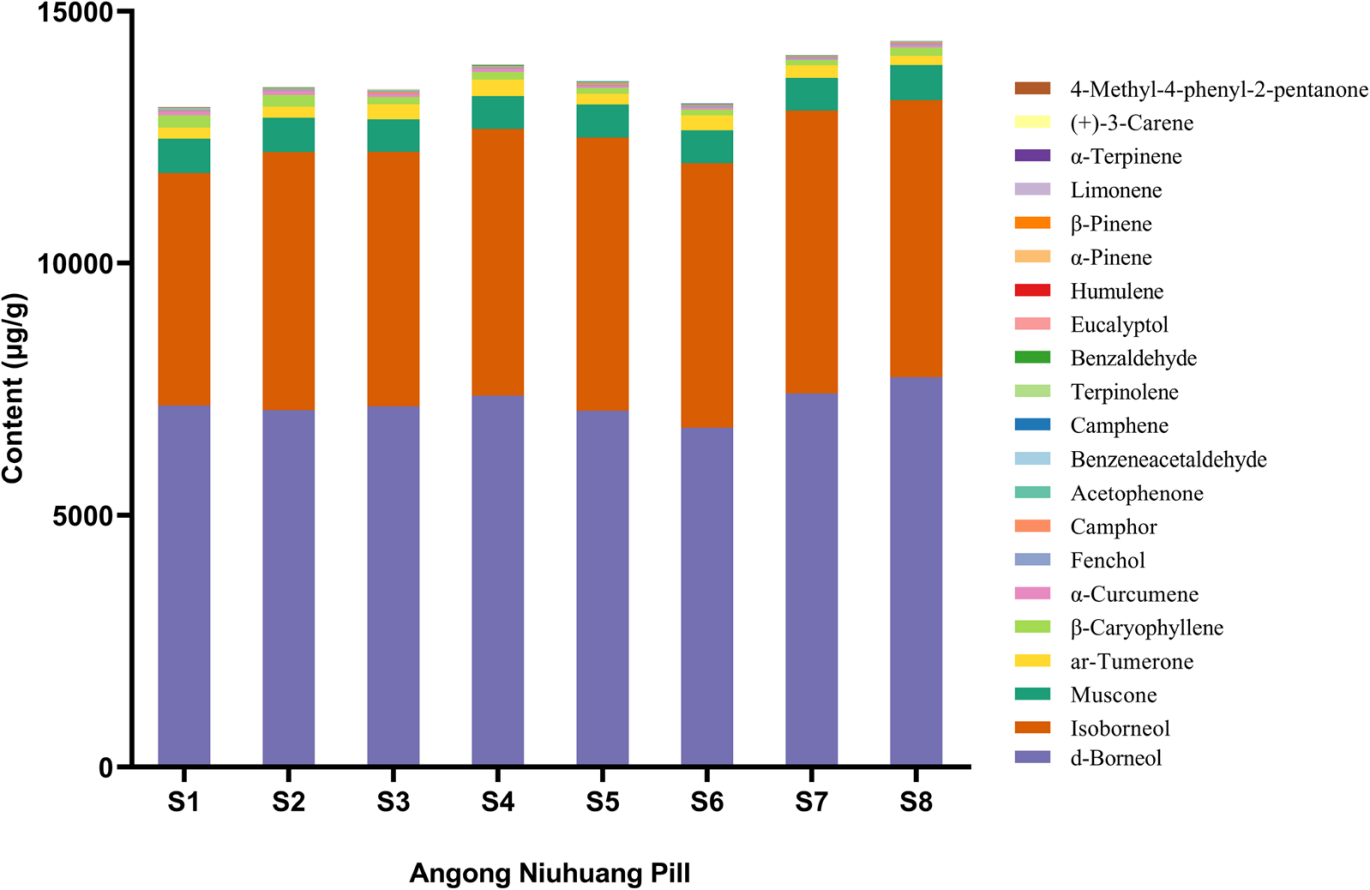
The content of 21 volatile compounds in 19 batches of ANP samples

In total, eight batches of ANP were analyzed by the developed and verified GC–MS/MS method, and the quantitative results are presented in Fig. 3 and Additional file 1: Table S5. It was found that d-borneol (6727.63–7734.92 μg/g), isoborneol (4614.45–5620.61 μg/g) and muscone (637.90–694.74 μg/g) were the top three abundant ingredients in all batches of ANP samples, making up 95% of the total contents at least. Notably, the characteristic ar-tumerone (186.33–324.22 μg/g), β-caryophyllene (106.81–244.95 μg/g), and α-curcumene (20.16–80.29 μg/g) were of high content level. The content of each component varied greatly, while the content distribution of each component in the eight batches was relatively consistent.

### Batch-to-batch similarity evaluation of ANP samples

**Fig. 4:**
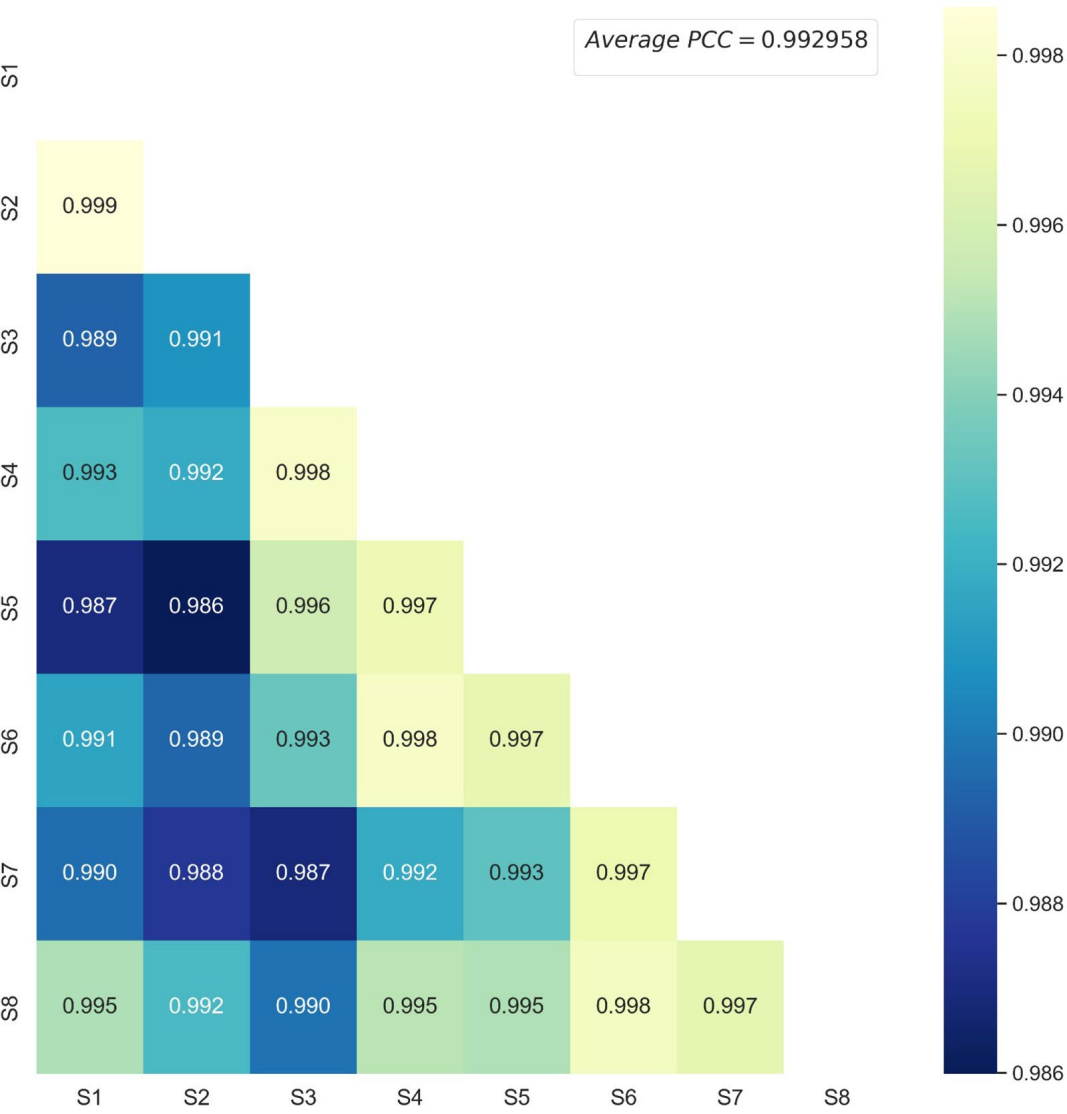
The heat map of Pearson correlation coefficients between 8 batches

In order to evaluate the batch-to-batch similarity of multiple ANP samples, the quantitative data of 8 batches were subjected to PCC analysis. For the sake of reducing the concentration distribution difference of different components, the concentrations were processed with logarithm first. Furthermore, the concentration heat map of eight batches was displayed in Additional file 2: Fig. S2, indicating that the concentration distribution of these batches are generally similar. Subsequently, the batch-to-batch similarity was quantified by constructing the correlation coefficient matrix, and the heat map of it was shown in Fig. 4. It could be found that the average PCC was 0.993, and even the lowest PCC between S2 and S5 was also more than 0.986, further revealing the high similarity among the eight batches.

### Characterization of volatile constituents of ANP in vivo

**Fig. 5:**
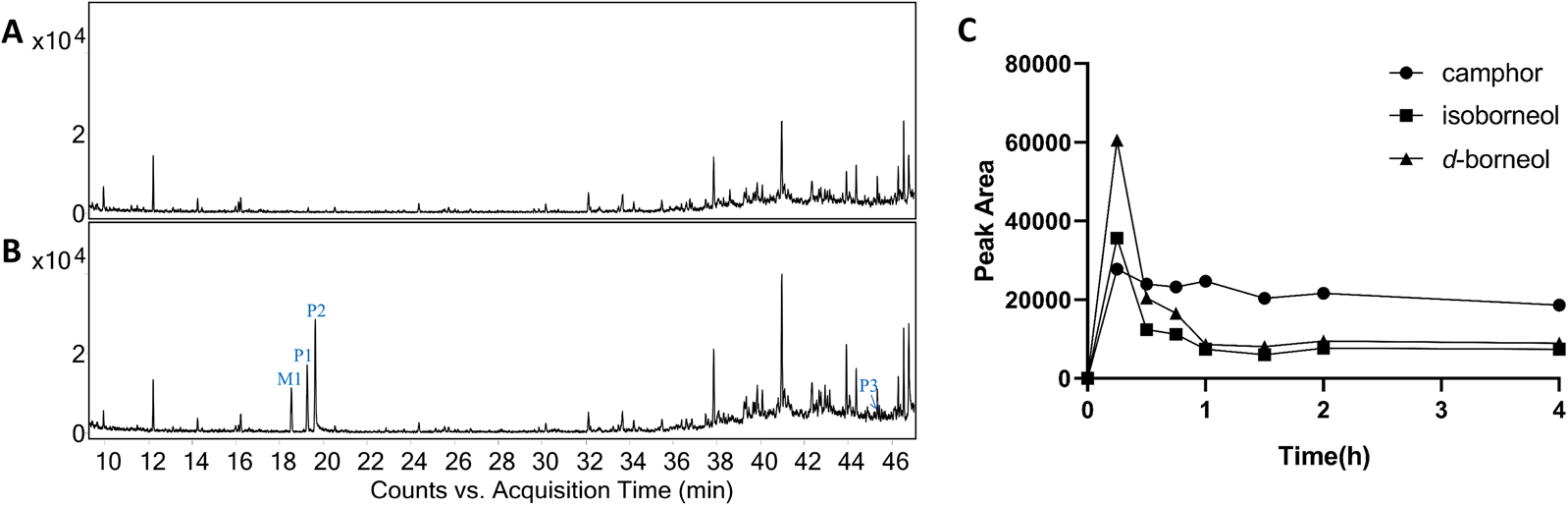
The total ion chromatograms of blank plasma sample (**A**) and administered plasma sample (**B**) at 0.25 h; **C** mean peak area-time curves for camphor, isoborneol and d-borneol in rats after oral administration of Angong Niuhuang Pill (n = 3)

To clarify the absorbed volatile component in vivo, the rat plasma after oral administration of ANP were analyzed by the developed GC–MS method. Since ANP was a classic first-aid Chinese patent medicine, single administration was performed. As shown in Fig. 5A, B, four absorbed components from ANP were characterized in 0.25 h, including three prototypes (P1–P3) and one metabolite (M1). By comparison with the retention time and mass spectral data of the reference standards, M1, P1, P2, and P3 were assigned to camphor, isoborneol (**28**), d-borneol (**29**) and muscone (**75**), respectively (Table 2), and their relative plasma concentration profiling in 0.25 to 4 h were depicted in Fig. 5C. The relative plasma concentration profiling of muscone could not be displayed because the content of muscone was extremely low at 0.25–4 h. The volatile constituents of most two high content in ANP were quickly absorbed after oral administration, and then decreased rapidly. While the content of camphor, the 2-position oxide of isoborneol and borneol [20, 21], remained relatively stable within 4 h, suggesting that isoborneol and borneol may be gradually metabolized into camphor. Curiously, no metabolites of muscone were detected, which were consistent with previously reports [22, 23]. Additionally, few metabolites were characterized partly because that single administration not accumulated the drug in vivo.

**Table 2:** Identification of the absorbed constituents in rat plasma after oral administration of ANP by GC–MS

No	t_R_ (min)	Compound	Formula	CAS	Structure type	Source
M1	18.54	Camphor^a^	C_10_H_16_O	76-22-2	Monoterpenes	B
P1	19.27	Isoborneol^a^	C_10_H_18_O	124-76-5	Monoterpenes	B
P2	19.63	d-Borneol^a^	C_10_H_18_O	464-43-7	Monoterpenes	B
P3	45.32	Muscone^a^	C_16_H_30_O	541-91-3	Ketones	M

Sources: *B* Borneolum, *M* Moschus

^a^The identification was confirmed with reference standards

Most notably, isoborneol, d-borneol and muscone played significant roles in the treatment of cerebrovascular diseases, corresponding to the main efficacy of ANP. Dong et al. [12] found that isoborneol and d-borneol may improve the function of neurovascular units through anti-apoptotic and anti-inflammatory properties so as to exert their protective effects against cerebral ischemia injury. Moreover, borneol and muscone acted as absorption enhancers in the blood–brain barrier by increasing paracellular and transcellular transport [11, 24, 25]. Muscone have been reported that could mediate neuroprotective effects against cerebral ischemia by preventing oxidative stress and Ca^2+^ influx [10]. When combined with other drugs, they can effectively promote the penetration of drugs through the blood–brain barrier, which is of great significance for the development of treating cerebrovascular diseases.

## Conclusion

In this study, GC–MS and GC–MS/MS were used to conduct a comprehensive qualitative and quantitative analysis of the representative volatile constituents in commercial ANP. Combined with retention indices and reference standards, the chemical characteristics of volatile constituents in ANP were described comprehensively and systematically for the first time. A total of 93 volatile components, assigned to four aromatic Chinese medicine, were preliminarily identified in ANP. Among them, 21 volatile components were selected and accurately quantified within 15 min based on the established GC–MS/MS method, in which d-borneol, isoborneol and muscone were the top three abundant ingredients in the ANP. According to the contents of 21 determined constituents, 8 batches of ANP were generally chemical similar based on PCC analysis. In addition, d-borneol, isoborneol and muscone and one metabolite (camphor) were found in rat plasma after single oral administration, suggesting that these prototypes and their metabolites could be potential bioactive substances of ANP. Collectively, this study provides a comprehensive information of volatile component of ANP in vitro and in vivo, which may helpful for the quality evaluation and pharmacological research of ANP. Meanwhile, the non-volatile constituents were also important for its holistic efficacy, which need further investigated using the appropriate analytical method.

## Supplementary Information

**Additional file 1: Table S1.** The information on eight batches of commercially available ANP samples. **Table S2.** Dynamic multiple reaction monitoring parameters of all analytes and internal standard. **Table S3.** Calibration curves, LODs and LOQs of 21 volatile analytes in ANP samples. **Table S4.** Intra-day precision, inter-day precision, repeatability, stability and recovery of 21 volatile analytes in ANP samples. **Table S5.** Contents of 21 volatile analytes in ANP samples (μg/g, mean ± SD, n = 4).

**Additional file 2: Figure S1.** The sources of volatile constituents in ANP samples. **Figure S2.** The concentration heat map of 21 volatile analytes in ANP samples from eight batches.

## Data Availability

The datasets used and/or analyzed during the current study are available from the corresponding author on reasonable request.

## Abbreviations

ANP: Angong Niuhuang Pill
TCM: Traditional Chinese medicine
GC–MS: Gas chromatography coupled with mass spectrometry
GC–MS/MS: Gas chromatography coupled with tandem mass spectrometry
IS: Internal standard
S/N: Signal-to-noise ratio
LOQ: Limit of quantification
LOD: Limit of detection
RSD: Relative standard deviation
NIST: National Institute of Standards and Technology
PCC: Pearson correlation coefficient
dMRM: Dynamic multiple reaction monitoring
