# Supplementary material for: Comprehensive chemical profiling of volatile constituents of Angong Niuhuang Pill in vitro and in vivo based on gas chromatography coupled with mass spectrometry
Source: Chin Med. 2022 Sep 10;17:105. doi: 10.1186/s13020-022-00659-8 (PMC9464384; doi:10.1186/s13020-022-00659-8)
Supplement: Supplementary file 2 — Additional file 2: Figure S1. The sources of volatile constituents in ANP samples. Figure S2. The concentration heat map of 21 volatile analytes in ANP samples from eight batches. [file 13020_2022_659_MOESM2_ESM.docx]

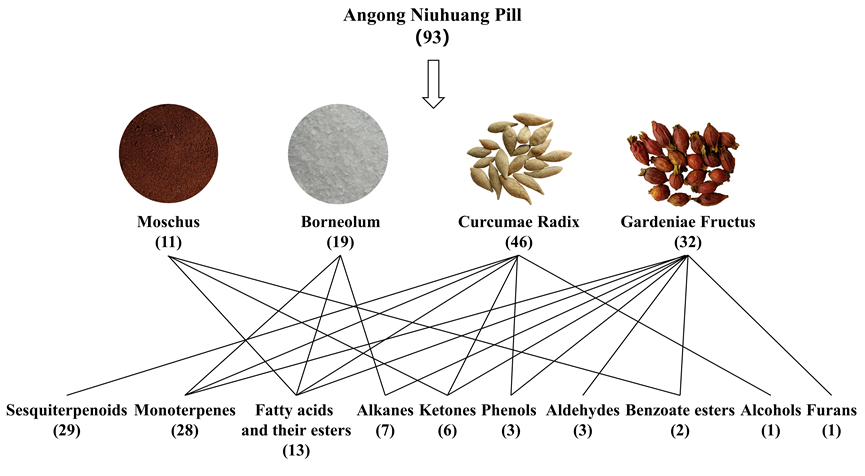


**Figure S1** The sources of volatile constituents in ANP samples.

**Figure S2** The concentration heat map of 21 volatile analytes in ANP samples from eight batches.
